# Seroprevalence and risk factors of *Toxoplasma gondii* infection in primary school children in Henan province, central China

**DOI:** 10.1051/parasite/2020018

**Published:** 2020-04-07

**Authors:** Shuai Wang, Zhijun Yao, Haoran Li, Pengju Li, Dong Wang, Haizhu Zhang, Qing Xie, Zhenchao Zhang, Xiangrui Li

**Affiliations:** 1 Xinxiang Key Laboratory of Pathogenic Biology, School of Basic Medical Sciences, Xinxiang Medical University Xinxiang 453003 Henan PR China; 2 MOE Joint International Research Laboratory of Animal Health and Food Safety, College of Veterinary Medicine, Nanjing Agricultural University Nanjing 210095 Jiangsu PR China

**Keywords:** *Toxoplasma gondii*, Primary school children, Seroprevalence, Risk factors, Central China

## Abstract

*Toxoplasma gondii* is an obligate intracellular protozoan parasite with global distribution. However, data on *T. gondii* infection among children in primary school in Henan province, central China were lacking. In this study, 2451 serum samples of primary school children in this province were collected from September 2015 to October 2018 and evaluated for *T. gondii* antibodies using an enzyme-linked immunosorbent assay (ELISA). The overall seroprevalence was 9.51% (233/2451), of which 7.59% (186/2451) showed IgG positivity, 0.73% (18/2451) IgM and 1.18% (29/2451) both. The main risk factors related to *T. gondii* infections were the age of children, residence area, contact with cats, and exposure to soil. Moreover, hand washing before eating was considered a protective factor. Seroprevalence of *T. gondii* infection among the study population was common, emphasizing the need to prevent and control this infection. This is the first report of *T. gondii* seroprevalence in primary school children in Henan province, central China.

## Abstract (fr)

*Toxoplasma gondii* est un parasite protozoaire intracellulaire obligatoire à distribution mondiale. Cependant, des informations concernant l’infection à *T. gondii* chez les enfants des écoles primaires n’étaient pas disponibles dans la province du Henan, en Chine centrale. Dans cette étude, 2451 échantillons de sérum d’élèves du primaire de cette province ont été collectés de septembre 2015 à octobre 2018 et évalués pour les anticorps contre *T. gondii* à l’aide d’un dosage immuno-enzymatique (ELISA). La séroprévalence globale était de 9,51 % (233/2451), dont 7,59 % (186/2451) représentaient la positivité des IgG, 0,73 % (18/2451) représentaient les IgM et 1,18 % (29/2451) représentaient les deux. Les principaux facteurs de risque liés aux infections à *T. gondii* étaient l’âge des enfants, la zone de résidence des enfants, les contacts avec les chats et l’exposition au sol. De plus, le lavage des mains avant de manger est considéré comme un facteur protecteur. La séroprévalence contre *T. gondii* dans la population étudiée était courante, ce qui souligne la nécessité de prévenir et de contrôler cette infection. Il s’agit du premier signalement de la séroprévalence contre *T. gondii* chez les enfants des écoles primaires de la province du Henan.

## Introduction

*Toxoplasma gondii* is a worldwide protozoan parasite that can infect virtually all warm-blooded animals, including humans. It has been estimated that one third of the world’s population has been infected with *T. gondii* [24]. The main route for humans to become infected with *T. gondii* is ingestion of raw or undercooked meat containing *T. gondii* tissue cysts from intermediate hosts [5, 16, 17]. In addition, humans can be infected through consumption of food or water contaminated with sporulated *T. gondii* oocysts [22], and vertical transmission during pregnancy from an infected mother to her fetus [3]. *Toxoplasma gondii* infection in healthy individuals is usually asymptomatic or only shows self-limiting flu-like disease, but blindness and intellectual disability can be caused in congenitally infected children and serious complications in immunocompromized patients, such as AIDS, cancer, and transplant patients [1, 15, 21]. Children and young adults can also develop toxoplasmic chorioretinitis, which is a common manifestation of congenital or acute infection [20].

Although the seroprevalence of *T. gondii* infection in primary school children has been reported all over the world [8, 9, 12], little is known about the seroprevalence of *T. gondii* infection in primary school children in China (Table 1; [10, 11, 18, 26, 29, 31]). Additionally, most of the articles reporting these data were published in local Chinese Journals in Chinese, and are not readily accessible to international readers. Moreover, reports on *T. gondii* seroprevalence among children in primary school in Henan province, central China are still lacking. Therefore, the objective of the present investigation was to examine *T. gondii* seroprevalence and relevant risk factors among primary school children in Henan province, central China.

**Table 1:** Prevalence of *T. gondii* infection in primary school children in the PR China.

Provinces/Cities	Year of sampling	No. of tested	No. of positive	Prevalence (%)	Method^a^	Reference
Nanchang, Jiangxi	1991	632	32	5.06	IHA, ELISA	[31]
Jiangmen, Guangdong	1995	18,408	586	3.18	ELISA	[26]
Boxing, Shandong	2005	814	54	6.63	IHA, ELISA	[18]
Qiqihar, Heilongjiang	2006	357	19	5.32	ELISA	[10]
Shiyan, Hubei	2008	750	78	10.40	ELISA	[11]
Shandong	2012–2014	6000	961	16.02	ELISA	[29]

a IHA: indirect hemagglutination test; ELISA: enzyme-linked immunosorbent assay.

## Materials and methods

### Ethics statements

In the current study, all of the protocols obtained the approval of the Ethics Committee of the Xinxiang Medical University (reference no. 2015018).

### The study area

The region where we performed this study was Henan province, located in the central part of China, with a total area of 167,000 km^2^ and an approximate population of 106.01 million. The Yellow River flows through the middle section of Henan, which is seated located at north latitude 31°23′ – 36°22′ and east longitude 110°21′ – 116°39′. The area has a mainland monsoon-type climate, with mean annual precipitation and mean annual temperature of 532.5–1380.6 mm and 12.1–15.7 °C, respectively, and four distinctive seasons. Henan province has 17 cities and the capital is Zhengzhou.

### Sample collections

**Figure 1:**
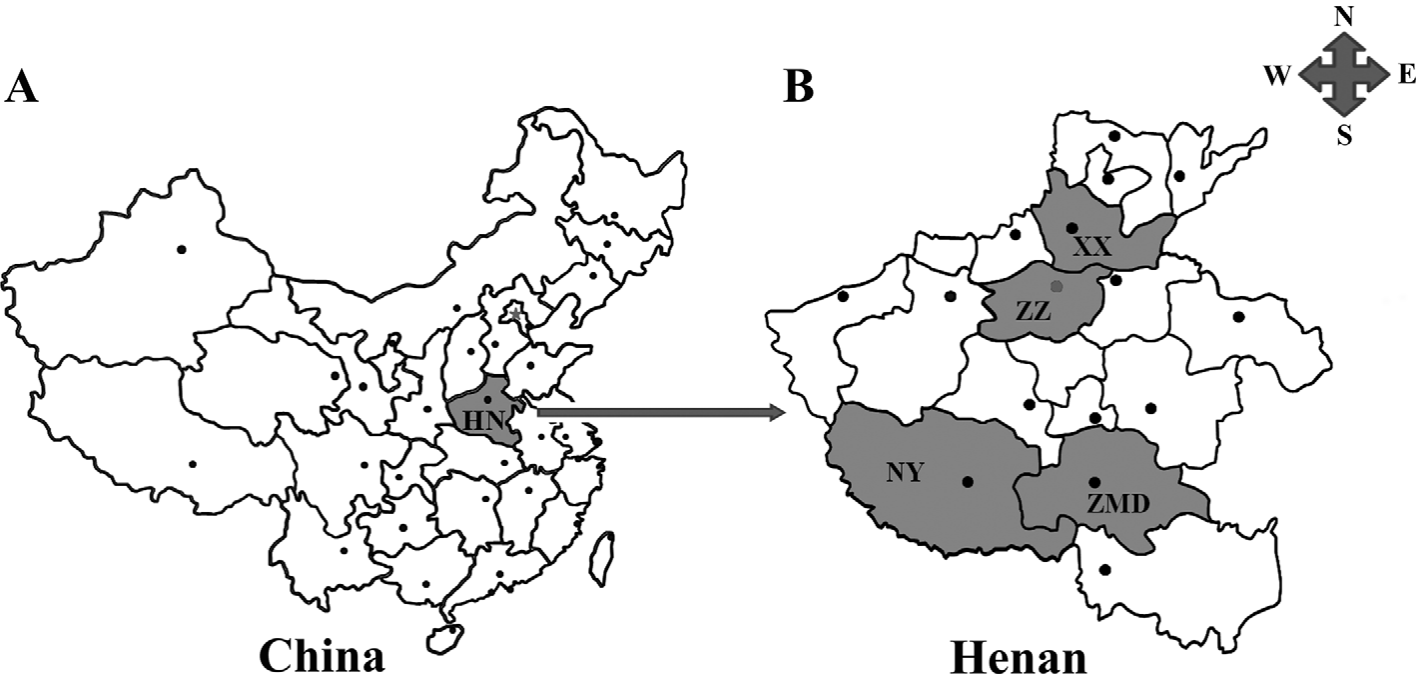
Geographic distribution of the sampling sites in Henan province, central China used in this study. (A) Henan province (HN, shadowed areas) is located in the central part of mainland China. (B) Shadowed areas are the sampling locations for the present survey. XX: Xinxiang; ZZ: Zhengzhou; NY: Nanyang; ZMD: Zhumadian.

In total, 2451 blood specimens were collected from primary school children aged from 6 years to 11 years participating in physical examinations and attending hospitals in four cities (Fig. 1) in Henan province from September 2015 to October 2018. Written informed consent was obtained from the parents/guardians of all included participants, after the objectives and procedures of this study were explained. A questionnaire was given to the parents/guardians of each child in order to gather information about risk factors, such as study region, sex, age, residential area, contact with cats, exposure to soil, and hand washing habits. Completed questionnaires were collected and stored for follow-up data analysis. All information collected was treated confidentially, and the data were coded and further analysed.

Blood samples were collected by well-trained nurses after obtaining assent from the participants and consent from their parents/guardians. Serum was separated by centrifuging blood samples and then transferred to 1.5 mL Eppendorf tubes for storage in a −80 °C freezer until testing for *T. gondii* antibodies.

### Antibodies to *T. gondii*

Antibodies (IgG and IgM) to *T. gondii* were detected using commercially marketed ELISA kits purchased from Zhuhai S.E.Z. Haitai Biological Pharmaceuticals Co., Ltd., Zhuhai, China. The detection procedure followed the manufacturer’s instructions. The sensitivity and specificity of IgG-ELISA were 92.9% and 97.3%, respectively [14]. The sensitivity and specificity of IgM-ELISA were 93.55% and 100.0%, respectively [28].

In brief, the test serum with a dilution ratio of 1:100 was added to each well in the coated plate and incubated at 37 °C for 30 min. After additional washing with washing solution, 50 μL peroxidase-conjugated anti-human immunoglobulin G (IgG) or IgM was added to the wells with incubation at 37 °C for 30 min, followed by three washings with washing solution. Color reactions were developed by adding 50 μL “A” solution and 50 μL “B” solution at 37 °C for 10 min, and then the stopping solution was added to stop the reaction. Microplates were read at an optical density (OD) of 450 nm in the MK3 microplate reader (Thermo Fisher Scientific, Waltham, MA, USA) and ratios (OD 450 nm value of serum sample/OD 450 nm value of negative control) were calculated after correction for the OD 450 nm value of the blank. The test serum samples were considered positive when the ratio was ≥2.1. The positive criterion for IgG and IgM was the same: a sample that was either IgG positive or IgM positive was considered a positive sample.

### Statistical analysis

Statistical analysis was performed using SPSS 20 software for Windows (SPSS Inc., Chicago, IL, USA). Statistical analyses of *T. gondii* prevalence in different variables were performed by *χ*^2^-test. Results were considered statistically significantly different if the *p-*value was less than 0.05.

## Results

As shown in Table 2, the overall seroprevalence of *T. gondii* among primary school children in Henan province was 9.51%; 7.59% (95% CI [6.54–8.64]) of children were positive for anti-*T. gondii* IgG antibodies only, 0.73% (95% CI [0.40–1.07]) of children were positive for anti-*T. gondii* IgM antibodies only, and 1.18% (95% CI [0.76–1.61]) of children were positive for both anti-*T. gondii* IgG and IgM antibodies.

**Table 2:** Seroprevalence of anti-*T. gondii* IgG and IgM antibodies in 2451 primary school children in Henan province, central China.

Antibodies	Absolute frequency	Relative frequency (%)	95% CI
IgG^+^, IgM^−^	186	7.59	6.54–8.64
IgG^−^, IgM^+^	18	0.73	0.40–1.07
IgG^+^, IgM^+^	29	1.18	0.76–1.61
IgG^−^, IgM^−^	2218	90.49	89.33–91.65
Total	2451	100.00	

+: positive; −: negative; CI: confidence interval.

According to Table 3, *T. gondii* seroprevalence among the children living in Xinxiang, Zhengzhou, Zhumadian, and Nanyang was 10.60%, 7.04%, 12.12%, and 8.53%, respectively. The prevalence of *T. gondii* antibodies varied significantly with the place of residence (*p* < 0.05). There was no significant difference in the seroprevalence of *T. gondii* between boys (9.78%, 95% CI [8.15–11.40]) and girls (9.21%, 95% CI [7.55–10.87]) (*χ*^2^ = 0.228, *p* = 0.633).

**Table 3:** *T. gondii* infection in primary school children in Henan province, central China.

Variable	No. of tested	No. of positive	Prevalence (%)	95% CI	*χ*^2^	*p*-value
Region						
Xinxiang	585	62	10.60	8.10–13.09	11.243	0.010
Zhengzhou	696	49	7.04	5.14–8.94		
Zhumadian	619	75	12.12	9.55–14.69		
Nanyang	551	47	8.53	6.20–10.86		
Sex						
Male	1289	126	9.78	8.15–11.40	0.228	0.633
Female	1162	107	9.21	7.55–10.87		
Age (years)						
6–7	819	62	7.57	5.76–9.38	7.254	0.027
8–9	784	74	9.44	7.39–11.49		
10–11	848	97	11.44	9.30–13.58		
Residence area						
Urban	1127	71	6.30	4.88–7.72	24.934	<0.001
Rural	1324	162	12.24	10.47–14.00		
Contact with cats						
No	1462	112	7.66	6.30–9.02	14.346	<0.001
Yes	989	121	12.23	10.19–14.28		
Exposure to soil						
No	659	36	5.46	3.73–7.20	17.131	<0.001
Yes	1792	197	10.99	9.55–12.44		
Hand washing before eating						
No	784	111	14.16	11.72–16.60	28.996	<0.001
Yes	1667	122	7.32	6.07–8.57		
Total	2451	233	9.51	8.35–10.67		

The overall *T. gondii* seroprevalence increased with increasing age (Table 3). In comparison to groups of 6–7-year-olds (7.57%) and 8–9-year-olds (9.44%), the *T. gondii* seroprevalence among older primary school children (10–11-year-olds) (11.44%, 95% CI [9.30–13.58]) was higher.

The seropositive rate of *T. gondii* in children living in rural areas was significantly higher than that in urban areas (*p* < 0.001). The seroprevalence of *T. gondii* in primary school children who had contact with cats was 12.23%, while 7.66% of primary school children who had no contact with cats were seropositive for *T. gondii.* Similarly, the probability that children became infected by *T. gondii* was increased by touching soil in comparison to no touching (10.99% vs. 5.46%, *χ*^2^ = 17.131, *p* < 0.001). Children with the behavior of washing hands before eating had lower seropositive rates for *T. gondii* than those without (7.32 vs. 14.16, *χ*^2^ = 28.996, *p* < 0.001).

## Discussion

*Toxoplasma gondii* infection in humans is widespread all around the world, and the prevalence varies in accordance with age, dietary habits, and environment [13, 30]. It was reported that the *T. gondii* prevalence in the Chinese population was about 7% during the two nation-wide surveys conducted in 1995 and 2004 [6], and increased up to 12.3% in 2010 [27].

It was revealed by this study that the total *T. gondii* seropositive rate was 9.51% in primary school children in Henan province, which was lower than the values of 16.02% among primary school children in Shandong province, eastern China [29], 14.4% in western Romania [4], 54.8% in the Republic of the Marshall Islands [9], and 63.1% in the Democratic Republic of São Tomé and Príncipe, West Africa [8]. The total *T. gondii* seropositive rate in Henan province was similar to the infection rate in Tehran, Iran (9.9%) [2] and Shiyan, China (10.4%) [11], but higher than that observed in Nanchang (5.06%) [31], Jiangmen (3.18%) [26], Boxing (6.63%) [18], and Qiqihar (5.32%) [10] in China. It is possible that various lifestyles, dietary habits, and geographical conditions are the most important factors underlying these differences, in addition to different investigational methods used.

In this study, male or female sex was not correlated with *T. gondii* seroprevalence, which was consistent with other reports [8, 9, 29].

It was indicated by previous studies that *T. gondii* seroprevalence was positively correlated with age of children [12, 23, 29], which also aligned with the results of the current study. It was hypothesized that this may result from increased exposure years as the child grows [12, 29].

Felids act as the only final host of *T. gondii* and thus play an essential role in transmitting this parasite. Infected cats are considered to be a potential threat to public health because they can excrete environmentally resistant oocysts in their feces [25]. In mainland China, seroprevalence of *T. gondii* infection in cats ranged from 3.9% to 79.4% [7]. The seropositive rate of *T. gondii* infection in domestic cats was 21.84% in Henan province [25]. Domestic cats are important companion animals of humans. In this study, contact with cats and touching soil have been found to be risk factors for children to exhibit *T. gondii* antibody positivity, consistent with the results of other studies [19, 23]. In the future, more research is needed to evaluate the prevalence of *T. gondii* oocysts in the soil of local parks and primary schools.

The seropositive rate of *T. gondii* in children living in rural areas was significantly higher than that in urban areas, which was consistent with other reports [4, 19]. Our previous research results confirmed that the seroprevalence of *T. gondii* in rural cats (29.26%) was significantly higher than in urban cats (16.35%) in Henan province [25]. This may lead to increased environmental contamination by *T. gondii* oocysts in rural areas, which in turn increases the risk of exposure to *T. gondii* in children. Additionally, hand washing before eating has been verified as a protective factor related to *T. gondii* seroprevalence in this study. This finding is in accordance with other similar surveys [19, 23].

## Conclusion

In conclusion, the results of this study suggest that *T. gondii* infection in primary school children is common in Henan province, central China. It is possibly helpful and necessary to take preventive actions like preventing soil from being contaminated by feces of infected cats, and educating children to wash hands before eating in this area.

## Conflict of interest statement

We declare that we have no conflict of interest.
